# Disease Specific Productivity of American Cancer Hospitals

**DOI:** 10.1371/journal.pone.0121233

**Published:** 2015-03-17

**Authors:** Jeffery A. Goldstein, Vinay Prasad

**Affiliations:** 1 Department of Pathology, Microbiology, and Immunology, Vanderbilt University Medical Center, Nashville, Tennessee, United States of America; 2 Bloomberg School of Public Health, Johns Hopkins University, Baltimore, Maryland, United States of America; University of Utah School of Medicine, UNITED STATES

## Abstract

**Context:**

Research-oriented cancer hospitals in the United States treat and study patients with a range of diseases. Measures of disease specific research productivity, and comparison to overall productivity, are currently lacking.

**Hypothesis:**

Different institutions are specialized in research of particular diseases.

**Objective:**

To report disease specific productivity of American cancer hospitals, and propose a summary measure.

**Method:**

We conducted a retrospective observational survey of the 50 highest ranked cancer hospitals in the 2013 US News and World Report rankings. We performed an automated search of PubMed and Clinicaltrials.gov for published reports and registrations of clinical trials (respectively) addressing specific cancers between 2008 and 2013. We calculated the summed impact factor for the publications. We generated a summary measure of productivity based on the number of Phase II clinical trials registered and the impact factor of Phase II clinical trials published for each institution and disease pair. We generated rankings based on this summary measure.

**Results:**

We identified 6076 registered trials and 6516 published trials with a combined impact factor of 44280.4, involving 32 different diseases over the 50 institutions. Using a summary measure based on registered and published clinical trails, we ranked institutions in specific diseases. As expected, different institutions were highly ranked in disease-specific productivity for different diseases. 43 institutions appeared in the top 10 ranks for at least 1 disease (vs 10 in the overall list), while 6 different institutions were ranked number 1 in at least 1 disease (vs 1 in the overall list).

**Conclusion:**

Research productivity varies considerably among the sample. Overall cancer productivity conceals great variation between diseases. Disease specific rankings identify sites of high academic productivity, which may be of interest to physicians, patients and researchers.

## Introduction

Academic productivity of individuals, institutions, and nations is widely measured, compared, and discussed [1], [2], [3]. [4]. In these measurements, two primary metrics are used 1) bibliometric, i.e. measuring publications or citations and 2) funding. Within academic medical centers, funding from the National Institutes of Health (NIH) (http://report.nih.gov/award/index.cfm), and the institutional h-index (a measure of publication and citations) have been used to boost morale, allocate resources, and judge leadership [4], [5], [6]. However, within the field of clinical cancer research, a broad overview of productivity is lacking.

The measurement of clinical trial productivity poses special problems. Clinical trials serve a dual role as vehicles for patient care and units of academic productivity. Cancer treatment, and therefore clinical research, is multifaceted, frequently involving surgical, medical, and radiological oncologists, as well as support from diagnosticians, general internists and surgeons. Specific diagnoses and their treatment rely on different specialists and subspecialists to differing degrees. The goal of this work is to provide an overview of clinical research productivity of leading academic cancer hospitals in the United States from 2008–2013, and to reflect the differences in productivity specific to particular diseases. Specifically, we hypothesize that different institutions specialize in research in particular diseases,.

## Materials and Methods

### Programming

Data acquisition and analysis were done using the Python programming language with the pandas, numpy, scipy, and matplotlib extensions. Please see below for a more detailed explanation of the programs’ functions. Code is available at https://github.com/jagstein/Rankings-dz

### Institutions

We used the US News and World Report top 50 hospitals in cancer. These listings are widely discussed and the overall scores and reputations have been reported to correlate with measures of academic productivity [7], [8]. Institutional groupings, e.g. Cornell University, New York Presbyterian Hospital, and Weill-Cornell Medical College were based on the US News rankings and extended to include relevant affiliated institutions. These affiliations are represented in the search terms and dictionary and presented in **S1–S2 Tables**.

### Published clinical trials and cumulative impact factor

Information on clinical trial publication and impact factor were determined by automated searching of PubMed using the BioEntrez and BioMedline packages and PubMed syntax (http://www.ncbi.nlm.nih.gov/books/NBK3827/, 6/1/2014). We considered 50 institutions and 27 diseases. For each institution / disease pair (e.g. Washington University (St. Louis) / urothelial cancers), we searched for published clinical trials, either all or restricted to Phase I or Phase II. We used the institutional and disease synonyms listed in **S1–S2 Tables**. For our over-all cancer results, we used the major MeSH category of cancer. As an example, the search for phase II cervical cancer clinical trials based at Washington University was formatted as:

(Barnes-Jewish Hospital[AD] OR Washington University[AD] OR Alvin J. Siteman Cancer Center[AD]) 2008:2013[DP] Clinical trial, Phase II[PT] uterine cervical neoplasms[MESH])

We counted the number of publications. For each publication, we identified the journal, and cross-referenced it with a published list of impact factors for 2012. We summed those impact factors. For example, if there were 3 trials published in journals with impact factors of 1, 2, and 3, respectively, the summed impact factor was 6.

### Clinical trials listed at Clinicaltrial.gov

We searched for all trials at clinicaltrials.gov with the search term 'cancer', yielding in 43,339 studies. These trials downloaded in XML format (5/24/2014), and provided data on the trial, start date, study id, phase of drug development, source of funding, number of participants, completion status, and lead institution. We performed an automated search through the trials. For each trial, we determined the disease(s) studied by reading in the title, conditions studied, and description, then searching for keywords specific to a particular disease, for instance, “urothelial cancer,” “bladder cancer,” and “ureteral cancer.” We searched each trial for each of the 27 diagnoses. For consideration of over-all cancer score, we used both classifiable and non-classifiable trials.

In this manner, 1 or more diseases were assigned to 31,164 trials. To check our assignments, we manually reviewed 100 of the most recently started trials for which no disease was identified. This check found 8 studies of our cancers of interest. Of the remaining 92 studies, there were 24 studies of side effects, e.g. mucositis, 15 of risk factors, e.g. psoriasis, 16 of cancers not covered, e.g. neuroblastoma, 13 of advanced solid cancers of unspecified type, 9 of non-cancer, non-risk factor conditions, e.g. diabetes, 5 basic research studies, e.g. drug-drug pharmacokinetic interactions between dabrafenib, rosuvastatin and midazolam, and 10 studies that could not be so grouped. We counted the number of trials in each phase that were administered by each institution. To handle institutions with multiple names, we combined institutions using a dictionary of institutions and common synonyms, which is presented as **S2 Table**.

### Statistical Analysis

To measure the degree to which clinical trial registration and clinical trial publication are redundant, we conducted regression analysis between two related measures, Phase II clinical trial registrations and Clinicaltrails.gov and Phase II clinical trial summed impact factors using the linear regression function from scipy. This function takes as input an array of values for x and one for y, and determines the slope, intercept, r value, p value, and standard error using a least-squares regression. We used the counts of registered phase II trials for the 50 institutions for each disease as the x and the summed impact factor of phase II trials for the same 50 institutions as the y. We ran a separate regression for the 25 diseases with non-trivial numbers of trials. The results for these regressions are shown in **S3 Table**. The slopes averaged 5.97+/-3.11 IF/registration (range 13.18–0.87), while the correlations (r2) averaged 0.328+/-0.183 (range 0.758–0.013). While this correlation was significant (p < 0.05) for 22 of 25, it is sufficiently low to justify consideration of both as independent factors in disease specific productivity.

We sought to create a summary measure of disease-specific academic productivity at particular institutions. We chose to focus on Phase II trials is based on the evidence of patient benefit from participation in these trials, as well as under-reporting of Phase I trials and the multi-centric nature of Phase III trials [9], [10]. While there are many measures of productivity based on publications, we sought to create a measure that accounted for clinical trial registrations as well. To this end, we generated a summary measure based on Phase II trials registered at Clinicaltrials.gov and the summed impact factor of Phase II clinical trials. This score for a given institution for a given disease was generated in the following manner:

$$\mathrm{Score}\left(\mathrm{institution},\;\mathrm{disease}\right)=\left(\frac{\mathrm{SIF}\left(\mathrm{institution},\mathrm{disease}\right)}{\mathrm{maxSIF}\left(\mathrm{disease}\right)}+\frac{\mathrm{Registrations}\left(\mathrm{institution},\mathrm{disease}\right)}{\mathrm{maxRegistrations}\left(\mathrm{disease}\right)}\right)\;*\;50$$

Where the SIF is the summed impact factor for Phase II trials, Registrations are the trials registered at Clinicaltrials.gov, maxSIF(disease) is the highest SIF among the 50 institutions for that disease, and maxRegistrations(disease) is the highest number of trials registered for that disease. This gives a maximum score of 100. For example, between 2008–2013, Barnes-Jewish Hospital published Phase II trials on cervical cancer with a summed impact factor of 7.993, and registered 1 Phase II trial at Clinicaltrials.gov. The University of Texas MD Anderson Cancer Center had the highest impact factor in cervical cancer at 10.329, while the University of Iowa Hospitals and Clinics registered the most trials, 2. Therefore, the score for Barnes-Jewish for cervical cancer is:

$$\begin{array}{c} \mathrm{Score}\left(\mathrm{Barnes}-\mathrm{Jewish},\;\mathrm{cervical cancer}\right)=\left(\frac{7.993}{10.329}+\frac{1}{2}\right)*50\\ =\left(0.774+0.5\right)*50\\ =63.69 \end{array}$$

## Results

### Overall productivity

We identified 6076 registered trials and 6516 published trials with a combined impact factor of 44280.4, involving 32 different diseases over the 50 institutions. For any disease under study at any institution, there were 11 different variables that could be measured, including 5 reflecting clinical trial registrations and 6 reflecting publication. The full data set is available in **S4–S5 Tables**. We calculated an overall cancer productivity score for each institution, with those results presented in **Table 1**.

**Table 1:** Overall cancer productivity.

Institution	All-cancer Score	Summed impact factor: Phase II, all cancers	Trial Registrations: Phase II, all cancers
**University of Texas MD Anderson Cancer Center**	100.00	2362.20	232
**Memorial Sloan-Kettering Cancer Center**	54.47	1168.19	138
**Dana-Farber/Brigham and Women's Cancer Center**	45.71	1009.09	113
**Massachusetts General Hospital**	26.98	562.10	70
**Johns Hopkins Hospital**	24.11	436.31	69
**University of Washington Medical Center**	20.79	208.45	76
**New York-Presbyterian University Hospital of Columbia and Cornell**	19.21	561.18	34
**Duke University Medical Center**	17.86	416.27	42
**Mayo Clinic Rochester**	17.02	539.51	26
**University of Michigan Hospitals and Health Centers**	15.94	284.78	46
**Moffitt Cancer Center**	15.27	273.38	44
**Ohio State University James Cancer Hospital**	15.15	400.13	31
**Barnes-Jewish Hospital/Washington University**	14.92	368.93	33
**UPMC-University of Pittsburgh Medical Center**	13.91	290.47	36
**Stanford Hospital and Clinics**	13.54	181.49	45
**Emory University Hospital**	12.00	210.44	35
**UCSF Medical Center**	11.69	165.18	38
**Roswell Park Cancer Institute**	10.39	144.58	34
**UCLA Medical Center**	9.96	165.09	30
**Northwestern Memorial Hospital**	9.77	176.54	28
**University of North Carolina Hospitals**	9.61	250.36	20
**University of Minnesota Medical Center**	9.58	238.69	21
**University of Wisconsin Hospital and Clinics**	9.49	81.76	36
**University of Chicago Medical Center**	8.91	146.23	27
**Vanderbilt University Medical Center**	8.63	275.19	13
**NYU Langone Medical Center**	8.48	217.50	18
**City of Hope**	8.07	116.71	26
**Cedars-Sinai Medical Center**	7.91	129.56	24
**Seidman Cancer Center at UH Case Medical**	7.79	82.88	28
**USC Norris Cancer Hospital**	7.74	19.29	34
**Oregon Health and Science University**	7.28	170.96	17
**Yale-New Haven Hospital**	6.08	114.38	17
**IU Health Academic Health Center**	5.95	87.41	19
**Fox Chase Cancer Center**	5.82	173.10	10
**Cleveland Clinic**	5.79	151.51	12
**Thomas Jefferson University Hospital**	5.06	218.84	2
**University of Colorado Hospital**	5.06	25.12	21
**Wake Forest Baptist Medical Center**	4.71	151.02	7
**Hospital of the University of Pennsylvania**	4.70	18.36	20
**University of Maryland Medical Center**	4.13	124.01	7
**University of Iowa Hospitals and Clinics**	4.13	72.81	12
**Beth Israel Deaconess Medical Center**	4.02	128.88	6
**Mayo Clinic Scottsdale**	3.88	0	18
**University of Kansas Hospital**	3.70	32.22	14
**UC San Diego Medical Center**	3.06	22.26	12
**Hackensack University Medical Center**	2.08	37.28	6
**Nebraska Medical Center**	1.94	9.96	8
**Hahnemann University Hospital**	0.43	0.00	2
**Mayo Clinic Jacksonville**	0.18	8.58	0
**Houston Methodist Hospital**	0	0	0

### Disease specific productivity

**Fig 1:**
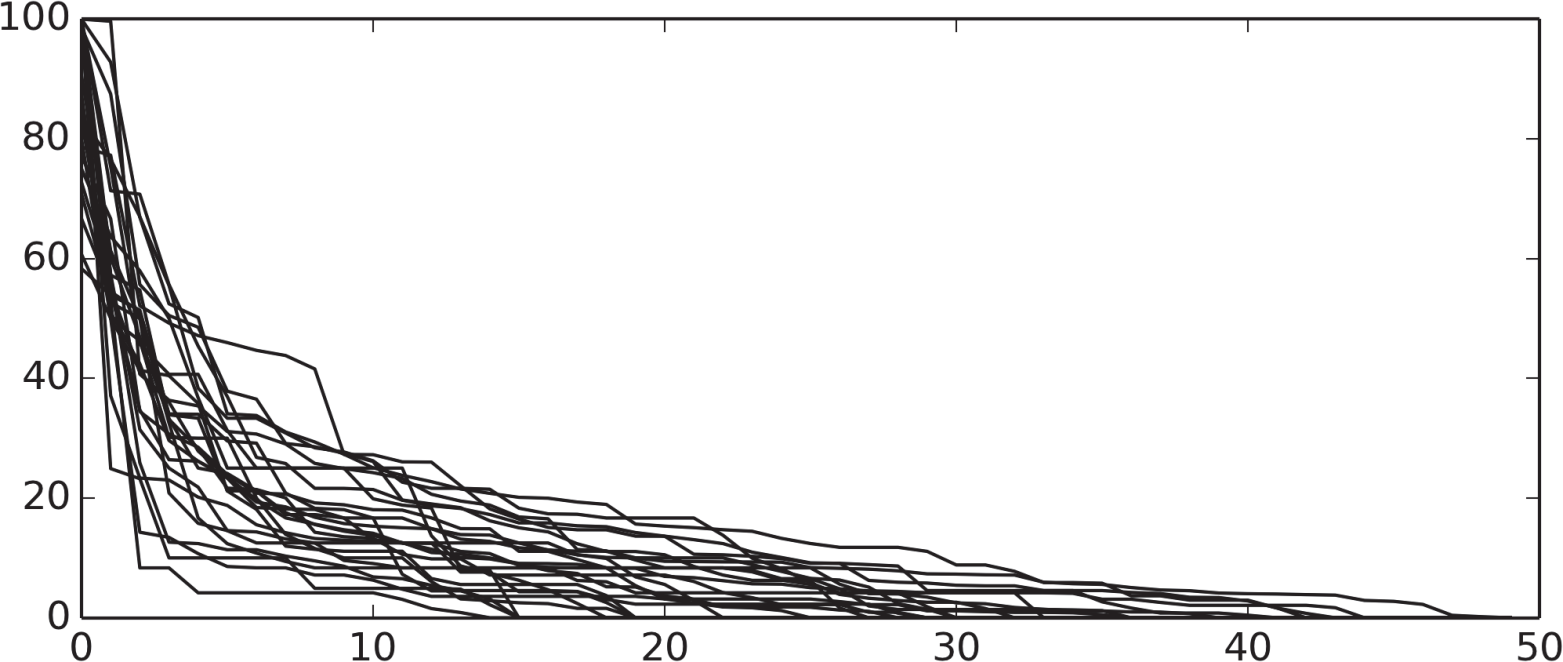
Rank-ordered scores for 25 cancer diagnoses, overlay. For most diseases the highest ranking institution (Rank = 1) has a score of 100, i.e. registering the highest number of clinical trials and publishing papers with the highest summed impact factor. As rank increases, the score rapidly declines, such that the institution with the 10^th^ highest score (Rank = 10) shows a score of 16.5 +/- 7.9 (average +/- standard deviation).

We collected publication and clinical trial data for each institution, produced disease-specific scores as described above and ranked them. There was minimal information on anal, vulvar, testicular, small intestinal, and penis cancers, so we did not analyze them further. We plotted the ranked scores from each disease and overlaid them (**Fig. 1**). For most diseases, one institution had the most clinical trials and the highest combined impact factor, for a score of 100. The scores for subsequently ranked institutions rapidly dropped.

### Cancer specific rankings

Different cancers are treated and studied by different physicians in different departments using a variety of techniques. To capture this diversity, we generated ranked lists over 25 different conditions. The 10 institutions with the highest score in each category, including ties, are presented as **Table 2**. M.D. Anderson Cancer center appeared on the most top-10 lists, 24/25, as well as having the highest score in 13/25. However, 43 of the 50 institutions make at least 1 appearance on a top 10 list, and 6 different organization were top ranked in at least one area. A full accounting of these appearances is presented as **Table 3**.

**Table 2:** Ranks by specific disease.

Condition	Institution	Score
**Acute Lymphocytic Leukemia**	University of Texas MD Anderson Cancer Center	85.71
	University of Washington Medical Center	50
	University of Minnesota Medical Center	20.57
	Thomas Jefferson University Hospital	14.29
	Memorial Sloan-Kettering Cancer Center	11.92
	Stanford Hospital and Clinics	10.71
	University of Chicago Medical Center	8.56
	Roswell Park Cancer Institute	8.34
	IU Health Academic Health Center	7.14
	Massachusetts General Hospital	7.14
	Oregon Health and Science University	7.14
**Acute Myelogenous Leukemia**	University of Texas MD Anderson Cancer Center	88.64
	University of Washington Medical Center	56.83
	Johns Hopkins Hospital	30.44
	University of Minnesota Medical Center	18.18
	Memorial Sloan-Kettering Cancer Center	14.67
	Stanford Hospital and Clinics	12.62
	Barnes-Jewish Hospital/Washington University	12.58
	Thomas Jefferson University Hospital	11.36
	Dana-Farber/Brigham and Women's Cancer Center	9.5
	Roswell Park Cancer Institute	9.09
**Biliary Cancer**	Massachusetts General Hospital	100
	Memorial Sloan-Kettering Cancer Center	58.72
	Ohio State University James Cancer Hospital	45.68
	University of Wisconsin Hospital and Clinics	40.67
	University of Texas MD Anderson Cancer Center	40.67
	New York-Presbyterian University Hospital of Columbia and Cornell	22.94
	University of Michigan Hospitals and Health Centers	16.67
	Roswell Park Cancer Institute	16.67
	Moffitt Cancer Center	16.67
	Stanford Hospital and Clinics	16.67
	Hospital of the University of Pennsylvania	16.67
	UCSF Medical Center	16.67
**Brain Cancer**	Duke University Medical Center	100
	University of Texas MD Anderson Cancer Center	58.18
	Dana-Farber/Brigham and Women's Cancer Center	40.9
	Massachusetts General Hospital	39.16
	UCLA Medical Center	30.98
	UCSF Medical Center	26.59
	Northwestern Memorial Hospital	22.93
	Memorial Sloan-Kettering Cancer Center	22.9
	Cedars-Sinai Medical Center	17.82
	University of Washington Medical Center	15.64
**Breast Cancer**	Dana-Farber/Brigham and Women's Cancer Center	100
	University of Texas MD Anderson Cancer Center	90.37
	Memorial Sloan-Kettering Cancer Center	71.14
	Barnes-Jewish Hospital/Washington University	51.46
	New York-Presbyterian University Hospital of Columbia and Cornell	49.18
	Emory University Hospital	35.38
	Johns Hopkins Hospital	32.32
	University of Kansas Hospital	29.4
	University of Washington Medical Center	28.95
	UCSF Medical Center	28.55
**Cervical Cancer**	University of Texas MD Anderson Cancer Center	75
	Barnes-Jewish Hospital/Washington University	63.69
	Dana-Farber/Brigham and Women's Cancer Center	57.97
	University of Iowa Hospitals and Clinics	50
	University of Minnesota Medical Center	36.74
	Seidman Cancer Center at UH Case Medical	25
	New York-Presbyterian University Hospital of Columbia and Cornell	25
	UC San Diego Medical Center	25
	Ohio State University James Cancer Hospital	25
	USC Norris Cancer Hospital	25
	Hospital of the University of Pennsylvania	25
**Chronic Lymphocytic Leukemia**	University of Texas MD Anderson Cancer Center	100
	Ohio State University James Cancer Hospital	50.98
	University of Washington Medical Center	43.33
	Mayo Clinic Scottsdale	16.67
	UC San Diego Medical Center	16.67
	New York-Presbyterian University Hospital of Columbia and Cornell	16.01
	Mayo Clinic Rochester	15.78
	Massachusetts General Hospital	15.39
	University of Wisconsin Hospital and Clinics	10.66
	Thomas Jefferson University Hospital	10
	Moffitt Cancer Center	10
**Chronic Myelogenous Leukemia**	University of Texas MD Anderson Cancer Center	95.83
	University of Washington Medical Center	53.07
	Thomas Jefferson University Hospital	8.33
	Roswell Park Cancer Institute	8.33
	Memorial Sloan-Kettering Cancer Center	4.17
	IU Health Academic Health Center	4.17
	UCSF Medical Center	4.17
	University of Chicago Medical Center	4.17
	Nebraska Medical Center	4.17
	Mayo Clinic Scottsdale	4.17
	Seidman Cancer Center at UH Case Medical	4.17
**Colorectal Cancer**	Memorial Sloan-Kettering Cancer Center	99.64
	University of Texas MD Anderson Cancer Center	81.25
	Dana-Farber/Brigham and Women's Cancer Center	47.59
	Duke University Medical Center	40.7
	Roswell Park Cancer Institute	34.03
	City of Hope	31.17
	New York-Presbyterian University Hospital of Columbia and Cornell	30.49
	UPMC-University of Pittsburgh Medical Center	29.44
	Vanderbilt University Medical Center	29.04
	Northwestern Memorial Hospital	27.89
**Esophageal Cancer**	Memorial Sloan-Kettering Cancer Center	100
	Dana-Farber/Brigham and Women's Cancer Center	60.2
	New York-Presbyterian University Hospital of Columbia and Cornell	59.39
	Johns Hopkins Hospital	37.5
	University of Texas MD Anderson Cancer Center	33.28
	Barnes-Jewish Hospital/Washington University	25
	Ohio State University James Cancer Hospital	24.11
	Fox Chase Cancer Center	17.57
	UPMC-University of Pittsburgh Medical Center	15.57
**Gastric Cancer**	Memorial Sloan-Kettering Cancer Center	100
	Dana-Farber/Brigham and Women's Cancer Center	57.4
	New York-Presbyterian University Hospital of Columbia and Cornell	43.94
	Roswell Park Cancer Institute	25
	Hospital of the University of Pennsylvania	21.82
	NYU Langone Medical Center	14.55
	Yale-New Haven Hospital	12.5
	Johns Hopkins Hospital	12.5
	Massachusetts General Hospital	12.5
	Fox Chase Cancer Center	12.5
	UPMC-University of Pittsburgh Medical Center	12.5
	Seidman Cancer Center at UH Case Medical	12.5
	Stanford Hospital and Clinics	12.5
**Head And Neck Squamous Cell Cancer**	University of Texas MD Anderson Cancer Center	100
	University of Chicago Medical Center	69.05
	UPMC-University of Pittsburgh Medical Center	68.84
	Memorial Sloan-Kettering Cancer Center	50.1
	University of Michigan Hospitals and Health Centers	42.61
	University of North Carolina Hospitals	28.57
	Dana-Farber/Brigham and Women's Cancer Center	27.16
	Johns Hopkins Hospital	21.65
	Mayo Clinic Rochester	21.43
	Duke University Medical Center	20.42
**Hodgkin's Lymphoma**	University of Texas MD Anderson Cancer Center	100
	Memorial Sloan-Kettering Cancer Center	42.94
	Massachusetts General Hospital	41.67
	Barnes-Jewish Hospital/Washington University	26.79
	Thomas Jefferson University Hospital	25
	Duke University Medical Center	25
	University of Minnesota Medical Center	16.67
	Ohio State University James Cancer Hospital	12.25
	City of Hope	9.91
	New York-Presbyterian University Hospital of Columbia and Cornell	8.33
	Northwestern Memorial Hospital	8.33
	IU Health Academic Health Center	8.33
**Liver Cancer**	Johns Hopkins Hospital	76.58
	University of Texas MD Anderson Cancer Center	75
	Massachusetts General Hospital	58.66
	Memorial Sloan-Kettering Cancer Center	47.18
	UPMC-University of Pittsburgh Medical Center	34.55
	Northwestern Memorial Hospital	34.17
	New York-Presbyterian University Hospital of Columbia and Cornell	28.39
	University of North Carolina Hospitals	26.69
	UCSF Medical Center	25
	Emory University Hospital	25
**Lung Cancer**	University of Texas MD Anderson Cancer Center	100
	Memorial Sloan-Kettering Cancer Center	76.06
	Massachusetts General Hospital	71.33
	Dana-Farber/Brigham and Women's Cancer Center	56.8
	University of North Carolina Hospitals	43.49
	Johns Hopkins Hospital	35.43
	Moffitt Cancer Center	30.2
	Emory University Hospital	25.39
	UPMC-University of Pittsburgh Medical Center	23.32
	New York-Presbyterian University Hospital of Columbia and Cornell	21.62
**Melanoma**	Memorial Sloan-Kettering Cancer Center	100
	University of Texas MD Anderson Cancer Center	98.6
	Vanderbilt University Medical Center	55.67
	Moffitt Cancer Center	50.5
	UPMC-University of Pittsburgh Medical Center	48.53
	Massachusetts General Hospital	37.85
	UCSF Medical Center	36.51
	University of Washington Medical Center	29.12
	Dana-Farber/Brigham and Women's Cancer Center	28.56
	Yale-New Haven Hospital	27.48
Myeloma	Dana-Farber/Brigham and Women's Cancer Center	62.5
	University of Texas MD Anderson Cancer Center	53.88
	University of Washington Medical Center	51.39
	Memorial Sloan-Kettering Cancer Center	42.39
	Mayo Clinic Rochester	33.99
	University of Michigan Hospitals and Health Centers	29.53
	NYU Langone Medical Center	29.17
	Mayo Clinic Scottsdale	20.83
	Massachusetts General Hospital	20.83
	Duke University Medical Center	18.28
**Non-Hodgkin Lymphoma**	University of Texas MD Anderson Cancer Center	100
	University of Washington Medical Center	34.36
	Memorial Sloan-Kettering Cancer Center	23.98
	Mayo Clinic Rochester	23.01
	New York-Presbyterian University Hospital of Columbia and Cornell	21.43
	Massachusetts General Hospital	19.56
	Dana-Farber/Brigham and Women's Cancer Center	16.16
	City of Hope	15.06
	Stanford Hospital and Clinics	13.56
	Johns Hopkins Hospital	12.44
**Ovarian Cancer**	Dana-Farber/Brigham and Women's Cancer Center	77.11
	University of Texas MD Anderson Cancer Center	64.29
	Memorial Sloan-Kettering Cancer Center	32.07
	Massachusetts General Hospital	29.51
	Cedars-Sinai Medical Center	27.29
	University of Minnesota Medical Center	21.54
	Fox Chase Cancer Center	19.19
	USC Norris Cancer Hospital	18.87
	New York-Presbyterian University Hospital of Columbia and Cornell	18.43
	NYU Langone Medical Center	18.28
**Pancreatic Cancer**	University of Texas MD Anderson Cancer Center	78.57
	Johns Hopkins Hospital	54.65
	Dana-Farber/Brigham and Women's Cancer Center	40.68
	Moffitt Cancer Center	22.92
	Stanford Hospital and Clinics	22.83
	Massachusetts General Hospital	22.58
	University of Michigan Hospitals and Health Centers	20.92
	Barnes-Jewish Hospital/Washington University	16.85
	Yale-New Haven Hospital	16.03
	UPMC-University of Pittsburgh Medical Center	15.71
**Prostate Cancer**	Memorial Sloan-Kettering Cancer Center	81.82
	University of Texas MD Anderson Cancer Center	78.6
	Dana-Farber/Brigham and Women's Cancer Center	58.31
	University of Wisconsin Hospital and Clinics	52.11
	UCSF Medical Center	47.13
	University of Michigan Hospitals and Health Centers	45.96
	New York-Presbyterian University Hospital of Columbia and Cornell	44.67
	Johns Hopkins Hospital	43.81
	Duke University Medical Center	41.57
	Massachusetts General Hospital	31.83
**Renal Cell Cancer**	University of Texas MD Anderson Cancer Center	78.09
	Dana-Farber/Brigham and Women's Cancer Center	69.65
	Memorial Sloan-Kettering Cancer Center	59.63
	Cleveland Clinic	50
	Stanford Hospital and Clinics	14.96
	Massachusetts General Hospital	12.5
	Yale-New Haven Hospital	12.5
	Seidman Cancer Center at UH Case Medical	12.5
	Hospital of the University of Pennsylvania	10.29
	Beth Israel Deaconess Medical Center	8.34
**Thyroid Cancer**	University of Texas MD Anderson Cancer Center	62.5
	Memorial Sloan-Kettering Cancer Center	54.5
	Ohio State University James Cancer Hospital	46.97
	Hospital of the University of Pennsylvania	29.74
	Massachusetts General Hospital	29.5
	University of Wisconsin Hospital and Clinics	25
	Mayo Clinic Rochester	21.19
	University of Chicago Medical Center	17.24
	Barnes-Jewish Hospital/Washington University	17.24
	University of Michigan Hospitals and Health Centers	17
**Urothelial Cancer**	Memorial Sloan-Kettering Cancer Center	86.5
	Massachusetts General Hospital	50
	University of Texas MD Anderson Cancer Center	41.34
	University of Michigan Hospitals and Health Centers	40.45
	Hospital of the University of Pennsylvania	35.78
	IU Health Academic Health Center	21.17
	Cleveland Clinic	18.11
	Stanford Hospital and Clinics	11.97
	University of Iowa Hospitals and Clinics	11.43
	Emory University Hospital	11.11
	Fox Chase Cancer Center	11.11
	Moffitt Cancer Center	11.11
	USC Norris Cancer Hospital	11.11
**Uterine Cancer**	University of Texas MD Anderson Cancer Center	100
	Dana-Farber/Brigham and Women's Cancer Center	34.91
	Memorial Sloan-Kettering Cancer Center	23.22
	UPMC-University of Pittsburgh Medical Center	20
	Yale-New Haven Hospital	10
	University of North Carolina Hospitals	10
	Seidman Cancer Center at UH Case Medical	10
	Ohio State University James Cancer Hospital	10
	University of Minnesota Medical Center	4.91
	USC Norris Cancer Hospital	4.91
	University of Iowa Hospitals and Clinics	4.91

**Table 3:** Appearances by institution.

Institution	Top 10 appearances	Number of times top-ranked
**Barnes-Jewish Hospital/Washington University**	7	0
**Beth Israel Deaconess Medical Center**	1	0
**Cedars-Sinai Medical Center**	2	0
**City of Hope**	3	0
**Cleveland Clinic**	2	0
**Dana-Farber/Brigham and Women's Cancer Center**	17	3
**Duke University Medical Center**	6	1
**Emory University Hospital**	4	0
**Fox Chase Cancer Center**	4	0
**Hackensack University Medical Center**	0	0
**Hahnemann University Hospital**	0	0
**Hospital of the University of Pennsylvania**	6	0
**Houston Methodist Hospital**	0	0
**IU Health Academic Health Center**	4	0
**Johns Hopkins Hospital**	10	1
**Massachusetts General Hospital**	17	1
**Mayo Clinic Jacksonville**	0	0
**Mayo Clinic Rochester**	5	0
**Mayo Clinic Scottsdale**	3	0
**Memorial Sloan-Kettering Cancer Center**	22	6
**Moffitt Cancer Center**	6	0
**Nebraska Medical Center**	1	0
**New York-Presbyterian University Hospital of Columbia and Cornell**	13	0
**Northwestern Memorial Hospital**	4	0
**NYU Langone Medical Center**	3	0
**Ohio State University James Cancer Hospital**	7	0
**Oregon Health and Science University**	1	0
**Roswell Park Cancer Institute**	7	0
**Seidman Cancer Center at UH Case Medical**	5	0
**Stanford Hospital and Clinics**	8	0
**Thomas Jefferson University Hospital**	5	0
**UC San Diego Medical Center**	3	0
**UCLA Medical Center**	1	0
**UCSF Medical Center**	7	0
**University of Chicago Medical Center**	5	0
**University of Colorado Hospital**	0	0
**University of Iowa Hospitals and Clinics**	3	0
**University of Kansas Hospital**	1	0
**University of Maryland Medical Center**	0	0
**University of Michigan Hospitals and Health Centers**	7	0
**University of Minnesota Medical Center**	6	0
**University of North Carolina Hospitals**	4	0
**University of Texas MD Anderson Cancer Center**	24	13
**University of Washington Medical Center**	9	0
**University of Wisconsin Hospital and Clinics**	4	0
**UPMC-University of Pittsburgh Medical Center**	9	0
**USC Norris Cancer Hospital**	4	0
**Vanderbilt University Medical Center**	2	0
**Wake Forest Baptist Medical Center**	0	0
**Yale-New Haven Hospital**	5	0
**Total institutions appearing at least once**	43	6

## Discussion

This paper describes the landscape of clinical research productivity in cancer and 25 of the most common specific diseases within highly ranked academic hospitals in the United States. The main finding is a granular description of what diseases are studied where.

Multiple scales of academic productivity have been proposed and utilized in an academic hospital setting, with varying focus on feasibility, validity, reliability and acceptability [11]. The institutional h-index, defined as h, where an institution has published at least h papers which have been cited at least h times has been used to compare academic departments between hospitals [5]. While papers published, number cited, impact factor, and h-index are predictive of future funding and future publication in academic surgery and neurosurgery departments, h-index was found to be superior to the other measures [6],[12]Since the description of the h-index in 2005, there have been numerous modifications proposed and difficulties identified (discussed in [12]). Funding has also been used, both as a measure of academic productivity and to validate the predictive value of other measures [5],[11].

While it is not our intention to propose *yet another* metric of research productivity for general use, the specific problems in the area of clinical trial productivity motivated our choice of measurements and summary measure. In terms of bibliometrics, Google Scholar, Web of Science, and Scopus, the 3 publication search engines that allow measurement of the h-index, do not allow restriction to clinical trials, or specific phases of clinical trials, a key feature of PubMed. Since publication of clinical trials makes up the minority of departmental output, this poses significant problems for their reliability. Similarly, the inability to restrict to MeSH terms means that a search for a particular cancer will identify some articles making comparisons to that cancer or discussing drugs used to treat that cancer. For example a search for “breast cancer” could return discussions of ovarian cancer or colon cancer due to the association of these diseases in the BRCA1 and 2 syndromes, or the use of trastuzumab (Herceptin, Roche/Genentech) for a variety of conditions, due to the primary indication of trastuzumab in the use of breast cancer over-expressing hER2. From a technical standpoint, automated PubMed searches can be conducted using the BioEntrez package in Python, while no similar capacity exists for the 3 proprietary databases.

Previous work has shown a high correlation between several different measures of academic productivity and USN&WR reputation[8]. We generated a composite score for each institution based on all phase II clinical trials registered at clinicaltrials.gov as well as the impact factor of phase II clinical trials published in MEDLINE. We present the overall scores compared to reputation as Table 1.

Several factors influence patient selection of a cancer hospital. Only 7.3% of patients seek care at an NCI-designated cancer center (NCI-CC)[13]. Although some groups have found an association between NCI-CC attendance and decreased mortality[14], patient characteristics differ. NCI-CC patients are younger, with fewer comorbidities and more advanced disease[13].

An obvious and validated factor in of hospital choice is distance [15]^,^[13]. For patients with treatment standards, who can expect a good outcome with standard-of-care treatment, the downsides of travelling farther may outweigh any benefits.

The benefit of treatment at an NCI-CC is thought to derive from improved process of care, potentially explaining the reduced mortality from both cancer and non-cancer causes[14]. There are also mortality improvements from seeking care at a high-volume facility[16]. We must accept the potential for confounding variables, as in the striking demographic and mortality differences that separate relatively well and well-off travelling patients from relatively ill patients for whom the NCI-CC happens to be their closest center[17]^,^[18].

Most of the institutions in this study are NCI-CC, and all have a high volume of cancer patients. Therefore, patients presenting to any of them can expect the benefits described above. However, defining the marginal benefit of seeking care at a higher ranked hospital is more difficult. The ‘Survival’ subscore given by US News for all of the top 50 hospitals is 8, 9, or 10. The weighting necessary to generate this score means that raters other than US News give different mortality scores for the same hospital[19]. This raises the question of whether any such measurements are feasible.

Our metric focuses on registration of phase II clinical trials and publishing them in high-impact journals. These activities differ from other academic ventures in that they involve potential benefit to patients. A review of phase II trials of molecularly targeted drugs indicated an average overall response rate of 6.4%[9]. This is consistent with the 4% response rate found more generally for phase I cancer trials[20]^,^[21]. This is a small degree of benefit, however it is attributable to the investigational agent and the investigator that administers it.

### Limitations

This analysis is subject to several objections and limitations. Errors of inclusion and exclusion of relevant trials are a potential concern; however, we performed manual checks in clinicaltrials.gov and PubMed on a limited number of unusual values. Automated and manual search both rely on proper initial curation of information. The error most likely to alter our rankings is failure to identify an institutional synonym, since this would present with an isolated drop of that institution. For that reason we present our list of synonyms and search terms (**S1–S2 Tables**). It might seem odd that, since these measures are so similar, the correlations between them display a wide range of values over different diseases. This speaks to the value of considering both measures since productivity may be missed if only one is considered. Clinical trial registration is forward looking, while publications are retrospective. Nonetheless, it is probable that some trials contribute to both components of our score. We would consider this as a positive since an institution that registers what it publishes and publishes what it registers is preferable to the alternatives.

We chose to focus on phase II trial counts from clinicaltrials.gov and impact factors of published phase II clinical trials from PubMed. We did not use NCI funding as a metric, because of the relatively low correlation with reputation, and the lack of direct patient benefit from the basic studies that form the bulk of NCI grants. We chose to focus on phase II trials because many phase I trials are not reported[10] while phase III trials tend to be multi-centric, and difficult to attribute excellence at the single-institution level. Patient counts in trials are heavily distorted by a small number of very large biobanking and prevention trials. Impact factor allows us to take into account the likelihood of a paper being read and cited. In an environment in which every clinical trial is increasingly expected to be published, small, poorly-designed, or less novel trials may be more likely to be published in lower tier journals.

### Conclusion

We provide a view of the landscape disease specific academic productivity in highly reputed American cancer hospitals. These hospitals show academic productivity among several diseases. Whether or not this translates into differences in patient care is unknown, and should be the subject of further study.

## Supporting Information

S1 Table Search terms. This table lists the disease-specific search terms used to classify trials. For clinical trial entries from Clinicaltrials.gov, the title, condition, and descriptive text of each trial was searched for each of the keywords (kw1–8), as well as the regular expression. The formatting of the regular expressions is such that, for example, ‘gastric.{1,100}cancer’ will detect any instances of the word ‘cancer’ within 100 characters after the word ‘gastric’, while ‘gastric cancer’ will only detect instances of that exact phrase. Detection of any of the keywords or the regular expression causes a trial to be classified as studying that disease. The MeSH terms are the terms used in PubMed with this ‘[mesh]’ tag to detect publications pertaining to that diagnosis.
(CSV)

S2 Table Institution dictionary. This is the list of sub-institutions that are combined in our analysis. For example, Barnes-Jewish Hospital, the Alvin J. Siteman cancer center (which is at Barnes-Jewish Hospital), and Washington University (which houses Barnes-Jewish Hospital) are all renamed Barnes-Jewish Hospital. In this case, Barnes-Jewish Hospital is used to reduce confusion with the University of Washington (Seattle). The synonyms were determined by manual search of the Clinicaltrials.gov database, the eponymous NCI cancer centers, and frequently encountered abbreviations.
(CSV)

S3 Table Clinicaltrials.gov – PubMed correlations. This list shows the correlation of the number of clinical trials registered at Clinicaltrials.gov and the summed impact factor of PubMed publications for specific diseases and for cancer overall.
(CSV)

S4 Table Clinical trial registrations by institution, disease, and phase. In combination with S5 Table, this represents a full accounting of the data underlying the summary measure used in the main body of the paper.
(CSV)

S5 Table Clinical trial publications and summed impact factors by institution, disease, and phase. (CSV)
